# African American College Students’ Drinking Behaviors and Their Relationship to Self-Efficacy and Positive or Negative Expectancies Regarding Alcohol Consumption

**DOI:** 10.3390/bs10100153

**Published:** 2020-10-06

**Authors:** Patrice R. Jenkins, Pedro M. Hernandez, Chaiqua A. Harris

**Affiliations:** 1 School of Social Work, College of Health Sciences, Jackson State University, Jackson, MS 39211, USA; pedro.m.hernandez@jsums.edu; 2 Department of Counseling, Rehabilitation, and Psychometric Services, Jackson State University, Jackson, MS 39217, USA; chaiqua.a.harris@jsums.edu

**Keywords:** alcohol consumption, positive expectancies, negative expectancies, college students, drinking behaviors, African Americans, historically Black colleges and universities

## Abstract

College students’ alcohol consumption remains a significant concern for colleges and universities. However, most research overwhelmingly utilizes White samples from predominantly White universities, limiting knowledge of African American students’ drinking behaviors on historically Black campuses. This study examined alcohol usage among African American college students by investigating relationships between alcohol consumption and positive and negative expectancies as well as self-efficacy. A convenience sample of 282 students was used. The Alcohol Use Disorders Identification Test (AUDIT) measured alcohol consumption and identified individuals whose consumption created hazardous drinking patterns. Alcohol expectancy was measured by the Alcohol Effects Questionnaire (AEQ), and the Spheres of Control Scale measured self-efficacy. Students in this sample tended to believe that alcohol consumption was linked with more negative than positive alcohol expectancy beliefs. Alcohol expectancies, specifically positive expectancies, appeared to play a significant role in predicting alcohol consumption. There was also a positive relationship between positive expectancies and alcohol consumption. Despite these results, our regression model was only able to account for about 20% of the variance (*r^2^* = 0.187). These findings are important in developing prevention and intervention programs to address the pervasive and critical social ills and reduce alcohol consumption among African American college students.

## 1. Introduction

Research indicates that as many as 9.6% of today’s college students self-report drinking habits that classify them as heavy drinkers (i.e., consuming 15 drinks or more per week), and when binge drinking (i.e., consuming five or more drinks in roughly two hours) is considered, these rates rise to 37% [1]. According to the National Institute of Alcohol Abuse and Alcoholism (NIAAA), 54.9% of full-time college students 18 and older reported drinking in the past month. These findings illustrate the continuing pervasiveness of alcohol consumption as a significant problem faced by colleges and universities across the country. Although drinking behavior on college campuses has been extensively investigated, most of these studies had a majority of White samples, limiting what is known about drinking behaviors on campuses of predominately African American colleges and universities, commonly known in the United States as Historically Black Colleges and Universities (HBCUs). The prevalence of problem drinking among college students and the severe health and social consequences associated with problem drinking mandate further research that facilitates understanding the problem at HBCUs.

Several questions have dominated the literature on problem drinking among African Americans, fueling research that examined the differences between the rates of drinking and problems related to drinking between African Americans and Whites, gender differences in drinking behavior among African Americans, and the social consequences of drinking among African Americans [2]. Although these factors are essential in understanding drinking behavior among African Americans, our study examines additional factors related to alcohol consumption, levels of self-efficacy, and alcohol expectancies among African American college students who drink. Self-efficacy is defined as a person’s personal beliefs in their ability to handle life situations; hence, their beliefs regarding their own personal drinking habits. Alcohol expectancies are defined as positive and negative attitudes regarding the outcome of alcohol usage. This study also examines alcohol use among African American college students, investigating possible relationships among alcohol consumption, levels of self-efficacy, and alcohol expectancies associated with drinking behaviors.

## 2. Literature Review

Historically, excessive drinking on college and university campuses across America has been the norm and remains a persistent problem. Undeniably, drinking is deeply rooted throughout campuses nationwide and has been one of the leading causes of death among college students. A 2019 report published by the NIAAA revealed that approximately 55% of students attending college drink. Thirty-seven percent reported drinking five or more drinks on occasion, a dangerous amount. Despite efforts to decrease drinking on college campuses, excessive alcohol consumption remains a national crisis. It is notably the leading cause of death among adolescents and emerging adults throughout this country [1].

A study conducted by Johnston et al. [3] revealed that compared to other age groups, young adults between the ages of 18 and 24 reported engaging more often in “risky” drinking behaviors than their counterparts. Notably, the highest prevalence of alcohol dependence occurs in this age group, regardless of whether they are in college. Hingson, Heeren, Winter, and Wechsler [4] found heavy drinking patterns peaking during late adolescence and early adulthood among 18- to 24-year-old college students. Timberlake et al. [5] showed that non-college-bound adolescents reported more heavy and binge drinking than those adolescents that entered college later on. However, the same study concluded that “college students not only surpassed their noncollege peers in alcohol use as young adults, but also exhibited a greater genetic influence on quantity of alcohol consumed per drinking episode” (p. 483).

Literature supports the notion that drinking patterns are established prior to entering college [5,6,7,8]. Students are arriving on college and university campuses having previously experimented with alcohol in high school. Miech et al. [7] found that by their senior year in high school, approximately 59% of students admitted to consuming alcohol, and 14% reported binge drinking in the past two weeks. Many college administrators have stated that schools across the country have been forced to inherit a problem without regard. However, administrations do not deny that the vast majority of students increase their consumption after arriving on campus [9]. Reports revealed that approximately 20% of college students in the United States met the diagnostic criteria for alcoholism, making campus prevention and treatment efforts a vital component of any effective anti-drinking campaign [1].

In efforts to curtail drinking patterns, colleges and universities have invested in advertisements and campus-wide campaigns against drinking. However, critics argue that campaigns as such are only a “band-aid fix” to a problem requiring reconstructive surgery, primarily due to traditional game-day tailgating parties, usually consisting of students (both underage and of age) drinking heavily [10]. Despite these efforts, over the last decade, college administrators have continued to adopt prevention programs aimed at on-campus alcohol use. In 2019, strides made by the NIAAA [11] resulted in the development of the Task Force on College Drinking. This Task Force included college presidents as well as research scientists. With its primary focus targeting the needs of college administrators and accumulating findings, the Task Force assisted in the establishment of individual-focused interventions. Based on their findings [8], recommendations were made to incorporate alcohol screenings as standard practices at college student health centers nationwide.

As mentioned earlier, the undeniable consequences of alcohol consumption have a negative impact on college students and individuals around them. Recent statistics published by the NIAAA [8] indicated the following drinking-associated consequences among students who drink:

- approximately 1519 deaths among students 18–24 years old;
- approximately 696,000 assaults on fellow peers;
- an estimated 97,000 sexual assaults or date rapes; and
- roughly 400,000 reported cases of unprotected intercourse.

The most severe consequence is alcohol poisoning, which is exacerbated by consuming 21 shots of alcohol within an hour [12]. Drinking on college campuses continues to be a major public health concern, but what is more disturbing is the lack of information regarding drinking behaviors among African American students. Research conducted around this topic has remained scant; thus, our findings will contribute to this body of knowledge.

The extent to which alcohol expectancies might be influenced by cultural socialization is only speculated based on the postulations of social cognitive theory (SCT). Founded by Albert Bandura [13] in the 1960s, SCT postulates that learning occurs in a cultural/social context with interconnectedness and correlation among the person, environment, and behavior. Particularly, emphasis on social influences and external and internal social reinforcements are unique constructs to consider when working with African Americans. Taking this into consideration, SCT focuses on ways individuals acquire and maintain specific behaviors. Reference is also given to one’s social environment and their behaviors within that environment. Influencing reinforcements, expectations, and expectancies, past experiences are key factors in determining whether a person will engage in a specific behavior and/or provide various reasons why an individual may engage in that behavior. Hence, the greater the expectancies, the more likely that students would consume alcohol. 

As mentioned earlier, speculations surrounding alcohol expectancies and the role culture plays remain unknown and warrant further research. In a recent study conducted by Braby, Holcomb, and Leonhard [14], the results revealed that students attending HBCUs exhibited lower rates of alcohol consumption and binge drinking compared to similar students from other universities nationwide. These lower levels of alcohol consumption among HBCU students could be connected to high scores on racial/ethnic identity and resiliency. Similarly, Zapolski and Clift [15] suggest that cultural socialization may decrease the risk of alcohol consumption among racial/ethnic minority youth. Other studies hypothesized that drinking frequency and quantity could be predicted by knowledge of alcohol expectancies [16,17]. There has also been a great deal of research demonstrating that positive alcohol expectancies may be important moderators in alcohol consumption [18,19,20]. Notably lacking in most of this research, however, have been substantial numbers of African American participants.

Nevertheless, articles cited in this literature review addressed many of the constructs usually advanced as moderators of drinking behaviors. However, the literature appears scant with respect to specific moderators that might be variables for college students, especially African American college students. Because of severe health risks related to alcohol use among college students, any confirmation of alcohol expectancies as predictors of drinking behavior would help develop prevention interventions [21].

Burke and Stephens [22] conducted a study exploring the role of social anxiety on drinking self-efficacy in college students. The study included 281 females and 159 male undergraduate students. The results revealed that, in situations that cause social anxiety, students with higher levels of social anxiety reported lower self-efficacy for avoiding heavy alcohol consumption when compared to students with low social anxiety. Lee and Oei [23] surveyed a “community sample of drinkers” and found that participants “who find it difficult to resist drinking when they are given opportunities to drink typically report higher frequency and quantity of alcohol consumption” (p. 385). Hasking and Oei [24] also found a significant interaction among alcohol expectancies and self-efficacy in predicting the amount of alcohol consumed in a community sample. The results revealed that lower drinking refusal self-efficacy yielded higher alcohol expectancies, which equated to higher levels of alcohol consumption; lower alcohol expectancies yielded lower levels of alcohol consumption. McBride, Barrett, Moore, and Schonfeld [25] reported that, among underage college students, positive alcohol expectancies were linked to higher prevalence of binge drinking. They found that two factors of their positive alcohol expectancies scale (i.e., sociability and sexuality) had significant effects on binge drinking.

Young, Connor, Ricciardelli, and Saunders [26] surveyed 174 undergraduate students, exploring the severity of alcohol dependence, frequency of drinking, and the quantity of alcohol consumed per occasion. The results revealed that positive alcohol expectancies showed significant variance in all three variables. However, negative expectancies were not predictors of drinking behaviors in this sample.

The results also revealed that “drinking refusal self-efficacy and dependence beliefs added additional variance over positive and negative expectancies in the prediction of all three drinking parameters” (p. 73). Young et al. [26] concluded that both positive expectancy and drinking refusal self-efficacy were strongly related to drinking among the population studied, and suggested that incorporating expectancy “as a means of informing prevention approaches” is necessary. Though this study presents variations among variables, the premise here is that drinking behaviors among college students continue to be a significant problem that warrants further research.

Although self-efficacy and outcome expectancies predicted alcohol consumption, Bandura’s ideology would support the notion that self-efficacy expectancies would be more salient than outcome expectancies in governing an individual’s behavior, suggesting, therefore, that expectancies and self-efficacy are salient variables in predicting drinking behaviors. Bandura’s [13] research provided scholars with the fundamentals for understanding the construct of self-efficacy. Self-efficacy was proven to be a key variable in the understanding of how thought affected an individual’s actions and behavioral patterns. Because of Bandura’s research, numerous studies have been conducted surrounding behavior and self-efficacy as they pertain to alcohol use. Though outcome expectancies and self-efficacy beliefs appeared to be equally important in predicting drinking behaviors, past research focused primarily on outcome expectancies [27,28,29,30]. However, Hasking and Oei [24] suggest that in recent years, researchers have become more interested in how beliefs concerning alcohol impact treatment outcomes. Brown [27] conducted a study measuring the effects of outcome expectancies on relapse. The results revealed a “consistent negative relationship between reinforcement expectancies, abstinence, and a number of alcohol-problem-free days in the subsequent year” (p. 128).

Although there has been extensive research surrounding alcohol consumption and experiences as moderators of consumption [17,18,19], the paucity of research, including a marginal sample size of African American college students attending HBCUs, remains an issue. The objective of the current study is to better understand the impact of drinking behaviors and alcohol expectancies in African American college students. It is our expectation that this study contributes to the body of literature on this subject.

## 3. Methods and Sampling

### 3.1. Participants

Data were collected from a convenience sample of 282 African American students who attended a historically Black urban university in the southern region of the United States. Students were recruited through word of mouth and flyers, and were offered an incentive for their participation in the study. The age range for the sample was 19 to 53 years of age, with a mean age of 23 (SD = 4.7). Ninety-eight percent (*n* = 276) of the participants were African Americans. The remaining 2% (*n* = 6) included one bi-racial individual and five individuals who self-identified as Africans. The sample consisted of 66% females (*n* = 187) and 34% males (*n* = 95). Table 1 provides a detailed summary of the sample characteristics. A power analysis was conducted to determine the sample size necessary to detect a small effect (*r* = 0.10–0.20, *p* < 0.05. Using Cohen’s power table, it was determined that a sample size of 125 would be necessary to detect a small effect. It is relevant to note that, after screening for missing data, the total sample size was reduced to 282.

### 3.2. Procedure

All procedures performed in studies involving human participants were in accordance with the ethical standards of the institutional and/or national research committee as well as with the 1964 Helsinki Declaration and its later amendments or comparable ethical standards. Protocol 0001-04 was approved by the Institutional Review Board at Jackson State University (FWA 00002620) IRB registration number 0005513.

Flyers were used as a form of recruitment and could not be distributed until the Division of Student Life granted approval. Informed consent was obtained from all individual participants involved in the study. The survey took an average of 30–45 min to complete. Students were instructed to consult the researcher if they did not understand the directions and/or the questions. Data were collected over four days (twice a week for two weeks) during the lunch hour.

### 3.3. Measures

Self-Efficacy: This variable was measured using the Spheres of Control Scale [31]. This scale is a 30-item instrument that explains perceptions of control within separate subscales. Respondents’ answers are gauged on a seven-point Likert-type scale indicating their degree of agreement for each of the 30 items. The Spheres of Control subscales measure three behaviors: interpersonal control (control over other people in dyads and groups), personal efficacy (control over the nonsocial environment, such as in personal achievement), and perceived sociopolitical control (control over social and political events and institutions). This measure was designed to assess these areas of control among college students. Each subscale is assessed using ten items. A sample item from the interpersonal control scale is “I have no trouble making and keeping friends.” A personal efficacy sample would reflect, “When I get what I want, it’s usually because I worked hard for it.” A third sample item from the sociopolitical control subscale is, “By taking an active part in political and social affairs we, the people, can control world events.” Response options range from 1 = agree to 7 = disagree. The possible range of scores for each subscale is 10 to 70. Higher scores will indicate perceived internal control. To test the reliability of the scale, reliability statistics were calculated. Cronbach’s alpha (i.e., a measure of internal consistency) for this scale was 0.825. The mean score was 131.66, with a standard deviation of 20.89.

Alcohol Expectancy: Alcohol expectancy was measured using the 40-item Alcohol Effects Questionnaire (AEQ) developed by [32] as a brief method of measuring an individual’s positive and negative beliefs about their personal alcohol use. A revision and extension of Brown, Goldman, Inn, and Anderson’s [27] Alcohol Expectancy Questionnaire, the AEQ contains 40 true and false items that assess both undesirable and enforceable effects of alcohol. This particular scale plays an important role in personal perception related to initiating and maintaining alcohol use. The AEQ has also been known to explain expectancy factors involved in relapse.

The AEQ contains eight scales used for scoring: (1) Global Positive (POS), (2) Social and Physical Pleasure (SPP), (3) Sexual Enhancement (SEX), (4) Power and Aggression (AGG), (5) Social Expressiveness (SOC), (6) Relaxation and Tension Reduction (REL), (7) Cognitive and Physical Impairment (IMP), and (8) Careless Unconcern (CU). Table 2 refers to the positive and negative subscales used for scoring. Possible scores on the scale range from 0 to 40; the higher the score, the greater the alcohol expectancies. The lower the score on the AEQ, the lower the expectations are regarding the effects of alcohol.

Alcohol Consumption: Alcohol consumption was measured using the Alcohol Use Disorders Identification Test (AUDIT) [33]. Developed by the World Health Organization [34], the AUDIT is a 10-item measurement designed to identify individuals whose alcohol consumption is hazardous and whose drinking habits may lead to alcohol-related problems. The AUDIT has been proven to be psychometrically stable through test–retest and internal consistency, as well as content, criterion, and construct validities [33,34].

The AUDIT has three questions measuring the amount or the frequency of drinking, three questions measuring alcohol dependence, and four questions measuring problems caused by alcohol. For this study, researchers were only interested in representing the frequency aspect of alcohol consumption by this population. The question regarding alcohol consumption frequency is “How often do you have a drink containing alcohol,” with response options ranging from 0 = never to 4 = four or more times a week. Data reflective of this are included in Table 3.

Though developed on a non-clinical population, normative data are available for the AEQ. Allen and Wilson [35] and Young, Oei, and Knight [30] reported that reliability (test–retest) and validity (predictive and postdictive) analyses had been conducted, and confirmed the psychometric strengths of the AEQ. To ensure reliability for the AEQ in this study, reliability analyses revealed that Cronbach’s alpha for the entire scale was 0.936. The mean score was 12.75, with a standard deviation of 9.677 (see Table 4).

The Spheres of Control Scale was explicitly designed for college-age individuals. Prior research has shown the Spheres of Control Scale to be internally consistent, reliable, and correlated with alcohol use [31]. Paulhus and Van Selst [31] reported, “Typical alpha reliabilities for the subscales are 0.75–0.80 on cross-validation samples. Test–retest correlations at four weeks are above 0.90 and at six months are above 0.70 for all three subscales” (*p*. 1,033). However, it was not a compatible fit for the population currently being studied. Reliability statistics were also calculated for each subscale. Cronbach’s alpha for the personal efficacy subscale is 0.696. The mean score was 48.53, with a standard deviation of 8.225. After deleting several items (12, 14, 17, 20) from the interpersonal control, Cronbach’s alpha increased in reliability to 0.707. The mean score was 20.59, with a standard deviation of 7.022. Cronbach’s alpha of 0.697 was achieved for the sociopolitical control subscale after deleting items (20–22). The mean score was 32.65, with a standard deviation of 8.127. Table 5 provides a detailed description of this measurement.

## 4. Statistical Analysis

Data were screened for missing data, normality, homogeneity of variance, and outliers. The assumptions for this data screening revealed that the assumptions for conducting correlations and multiple linear regressions were met. Fifteen surveys were eliminated due to missing data. Various statistical procedures were performed using SPSS software [36]. Descriptive statistics were calculated on each variable, providing means and standard deviations. Bivariate correlation was calculated to evaluate the relationship between positive alcohol expectancies and alcohol consumption. Multiple linear regressions were utilized to determine which variables were predictors of alcohol consumption.

## 5. Results

Research question one queried as to whether students had more positive or negative alcohol expectancy beliefs. A frequency analysis was done to answer this question. The majority of the students (59.9%) did not endorse the Global Positive Expectancy subscale. An additional 16% endorsed only one of the positive expectancies, indicating that an overwhelming majority of 76% of the sample endorsed one or less of the positive expectancies. Conversely, 68% of the sample endorsed one or more negative expectancies on the Cognitive and Physical Impairment subscale, and 60% endorsed one or more negative expectancies on the Careless Unconcern subscale. These frequencies show that the students in this sample frequently believed that alcohol consumption has more negative than positive outcomes. The correlation between positive beliefs and drinking outcomes was statistically significant (*r* (267) = 0.366, *p* < 0.01). A chi-square test, x^2^ (267) = 235.998, *p* < 0.0001, indicated that the distribution of these variables was due to chance. Therefore, positive beliefs and alcohol consumption were definitively dependent on one another.

Research question two addressed whether a relationship existed among alcohol consumption, domains of self-efficacy, and alcohol expectancies. Multivariate regression was conducted to answer this question. Stepwise regression was used to understand the contribution of the domains of alcohol expectancies and self-efficacy to the model. As expected, there continued to be a strong positive correlation between alcohol consumption and alcohol expectancies. A partial relationship existed among alcohol consumption and the interpersonal control subscale. Frequency analysis revealed that most African American participants reported some level of drinking behavior, and 47% of those drinkers reported drinking more frequently than once a month. The regression results indicated that positive expectancies predicted levels of alcohol consumption (*r*² = 0.187, *r*²adj = 0.170, F(5, 236,) = 10.845, *p* < 0.01, *β* = 0.332, *t* = 4.228, *p* < 0.01). The results further revealed that self-efficacy alone did not predict levels of alcohol consumption. However, the interpersonal control subscale was the only level of the self-efficacy domain that proved to be a partial predictor of alcohol consumption. The more control an individual has over other people in dyads or groups, the less likely they are to drink heavily (*β* = 0.155, *t* = 2.153, *p* < 0.05; see Table 6).

## 6. Discussion

Drinking on college campuses has always been an area of great concern for college administrators. Colleges and universities nationwide are continuing to strategically incorporate plans for reducing alcohol consumption and alcohol-related problems among their students. This study examined alcohol use among African American college students, emphasizing possible relationships among alcohol consumption, domains of self-efficacy, and alcohol expectancies. The study sought to investigate further additional demographic and sociocultural factors associated with drinking behaviors in this population.

The results from the correlation analysis indicated a significant relationship between positive expectancies and alcohol consumption. Participants who had positive beliefs regarding drinking outcomes consumed higher levels of alcohol in this sample. Magri et al. [37] suggest that college students with high positive alcohol expectancies have a higher risk of suffering from an alcohol disorder. The researchers posit that, in keeping with expectancy theory, if a student believes that drinking alcohol will provide positive benefits, such as reducing anxiety and increasing their sociability, they will drink more in settings where alcohol is available. Expectancy theorists [14,34] also suggest that if this group of students were placed in an environment where alcohol was available, they would be more likely to drink. A valid explanation for this could lie in the premise that outcome expectancies provide a direct link between environmental factors and a person’s behavior.

Embedded in social cognitive theory, positive expectancies have proven to be a predictor of alcohol consumption among college students in general. As stated earlier, there has been an enormous amount of research examining the relationship between positive expectancies and drinking patterns [17,18,19]. However, lacking in these studies has been the presence of African American participants. These findings are significant because they provide insight into the behavior and drinking patterns of African American college students and, in this sample, indicate that they are not that different from the majority population.

Previous research [38] provided support for this finding and revealed that the amount of alcohol an individual consumed depended on the outcome they expected to gain from drinking. For example, if students expected positive rewards from consuming alcohol, they would drink in a pattern that maximizes their expected outcome. Additionally, a study by Young et al. [25] further validated the current findings of this study. In their surveying of 174 college students, the authors found positive expectancies to be a predictor of alcohol consumption. Our study asserts that positive alcohol expectancies are a significant variable in predicting the quantity of alcohol consumption for African American college students.

Interpreting the current findings would suggest that African American students who believe they are in control of their drinking will be more likely to drink in suitable situations. This same group of students is more likely to drive while under the influence or intoxicated because of the perception of control. There was no interaction between the domains of self-efficacy and alcohol expectancies. These African American students who drank felt that no one variable had to interact with the other to consume alcohol.

The findings revealed that positive beliefs regarding drinking outcomes were predictors of alcohol consumption among African American college students that participated in this study. The results from a multiple regression partially supported that domains of self-efficacy would be predictors of alcohol consumption among African American college students who drink. Namely, the interpersonal control subscale was the only level of the self-efficacy domain that proved to be a significant predictor of alcohol consumption. Specifically, students who have control over other people in dyads and groups were more likely to consume alcohol as a result of their perceived sense of control. Thus, self-efficacy was not a contributing factor in determining alcohol consumption. Though self-efficacy did not emerge as a contributing factor, Bandura [13] argued an association between self-efficacy and outcome expectancies. He further explained that self-efficacy beliefs accounted for the greatest variance in an individual’s behavior when the outcome depended on the behavior. Notably, students who generally display higher levels of self-efficacy might perceive themselves as being able to successfully master prospective situations that are often stressful.

An explanation for these results could also have relevance to Paulhus’s and Van Selst’s [31] idea that personal confrontations with society are decomposed into three distinctive spheres. People develop personal efficacy as a result of their achievements within a nonsocial environment. Once personal efficacy has been achieved, they (students) become more comfortable interacting within various groups and developing social relationships, thus maintaining interpersonal control. Perceptions are heightened as a result, and they are more apt to challenge political or social institutions whose ideologies conflict with theirs. Paulhus and Van Selst [31] also suggested that “personal efficacy, interpersonal control, and sociopolitical control are conceptually independent dispositions” (p. 1034). Individuals could have different expectations of control within their three distinct spheres of interaction with society.

Finally, a multiple linear regression analysis indicated that alcohol expectancies, solely positive expectancies, contributed to alcohol consumption. In the self-efficacy domain, only one of the three levels, that is, interpersonal control, showed a significant effect on alcohol expectancies. This outcome was unexpected because previous studies found a direct link between levels of self-efficacy and alcohol expectancies. These findings, in part, could be related to one’s own beliefs, thoughts, actions, and sense of control when consuming alcohol in various settings. Their level of control determines their thoughts and beliefs regarding when and how much they consume. Zapolski and Clift [15] reported that cultural socialization decreased the risk of alcohol consumption indirectly through alcohol expectancies among racial minority adolescents.

Hasking and Oei [23] found a significant interaction among alcohol expectancies and self-efficacy in predicting the amount of alcohol consumed in a community sample. The results revealed that lower drinking refusal self-efficacy yielded higher alcohol expectancies, which equated to higher levels of alcohol consumption; lower alcohol expectancies yielded lower levels of alcohol consumption. Lee and Oei [22] surveyed a “community sample of drinkers” and found that participants “who find it difficult to resist drinking when they are given opportunities to drink typically report higher frequency and quantity of alcohol consumption” (p. 385).

### Limitations and Future Research

There were several limitations noted in the study. Regarding the measurements utilized, the Spheres of Control measurement was explicitly designed for college-age individuals; however, this instrument was not validated with an African American student population. Reliability statistics provided a Cronbach’s alpha for the entire scale (0.825) and the personal self-efficacy subscale (0.696). Unfortunately, for the remaining two subscales, interpersonal control and sociopolitical control, an increase in Cronbach’s alpha was not achieved until the deletion of several items from each scale.

A second limitation involved the AEQ, a 40-item true-or-false instrument measuring an individual’s positive and negative beliefs regarding alcohol consumption within eight subscales. The AEQ was not psychometrically sound, and the eight subscales did not hold up when an attempt was made to extract them into the data set. Engels, Wiers, Lemmers, and Overbreek [39] also noted, “Although it is possible to employ all subscales of the AEQ to look at different associations with drinking in specific situations, the high intercorrelations between subscales of the AEQ make it difficult to conduct multivariate analyses in which the different aspects of alcohol expectancies would appear to be related to specific drinking patterns” (p. 161).

## 7. Implications for Social Work Practice

This research is relevant to social work practice and social work research in that it contributes to the overall knowledge base of the profession. The findings from this study also emphasize the continued need to develop interventions targeting college students with hazardous drinking habits. Although the NIAAA [40] continues to publish recommendations for elevating or decreasing the number of alcohol-related deaths and problems caused by alcohol abuse, the findings suggest that there is still work to be done. Realizing that these recommendations provide comprehensive strategies for integrating programs that foster student involvement with a focus on self-determination, it is important to keep in mind that students today are faced with multiple factors that could contribute to their drinking habits. Therefore, in developing prevention programs, it is necessary to create a plan that provides a holistic approach in addressing this population.

The literature review for this study revealed that the age of consumption and the amount consumed by high school students have proven to be strong predictors of heavy drinking in college. Based on current findings regarding interpersonal control and expectancies, it would be beneficial for policymakers to consider current guidelines and future ones regarding underage drinking. College administrators have vigorously shared concerns regarding incoming students who exhibit strong drinking patterns due to pre-exposure during high school. By making efforts to combat this problem, high school and college administrators have an opportunity to develop primary prevention programs targeting this specific group of students.

The findings concerning the relationship between alcohol expectancies and consumption can contribute to the development of interventions that could positively impact college students’ general health and well-being. Additionally, insight from this study provides opportunities to evaluate the effectiveness of current prevention programs as well as the establishment of new programs.

Because predictors of drinking behaviors change and research remains scarce about African American participants, one recommendation for future research would be to replicate this study at HBCUs across the state. This recommendation would provide additional information about the drinking behaviors and beliefs of African American students attending school in rural environments. Another recommendation would be to investigate possible gender differences and levels of alcohol consumption among this population. A final recommendation is to explore expectancies associated with the use and abuse of other substances among African American college students. Alcohol expectancies are essential factors in understanding why students drink in general.

The overall findings of this study indicated that African American college students might have problems with alcohol consumption; however, when compared to the consumption rates presented in the research literature on this topic, their consumption rates are much lower than those of students at predominantly White institutions. They are also lower than their African American counterparts attending predominantly White institutions. The findings also indicate that the relationships between the factors contributing to alcohol consumption in this population are complicated and warrant further study.

## Figures and Tables

**Table 1:** Summary of sample characteristics (*n* = 282).

Characteristics	*n*	%
*Gender*		
Men	95	33.7
Women	187	66.3
*Race*		
African American	276	97.9
African	5	1.8
Bi-racial	1	0.3
*Relationship*		
Single	261	92.5
Married	13	4.6
Separated	3	1.1
Divorced	5	1.8
*Employment*		
No	7	2.5
Yes	119	42.2
Part-time	122	43.3
Full-time	34	12.0
*GPA*		
Below 1.00	5	1.8
1.00–1.99	112	39.7
2.00–2.99	164	58.1
3.00–4.00	1	0.4
*Religion*		
Christian	266	94.3
Other	16	5.7
*Importance of Religion*		
Not Important	5	1.8
Not Too Important	4	1.4
Somewhat Important	19	6.7
Fairly Important	51	18.1
Very Important	203	72.0

*n* = frequencies; % = percentage.

**Table 2:** Summary of alcohol expectancy subscales.

Subscales	Item Number
*Positive Subscales*	
1. Global Positive 2. Social and Physical Pleasure 3. Sexual Enhancement 4. Power and Aggression 5. Social Expression 6. Relaxation and Tension Reduction	8, 17, 22, 29, 40 13, 15, 21, 24, 27 7, 12, 19, 28, 31 1, 5, 9, 16, 32, 37 3, 20, 35, 38, 39 2, 4, 11, 25, 34
*Negative Subscales*	
7. Cognitive and Physical Impairment 8. Careless Unconcern	6, 10, 18, 23, 26 14, 30, 33, 36

**Table 3:** Summary of alcohol drinking frequencies.

How Often Do You Drink Alcohol?	*n*	*%*
Never	67	23.8
Monthly or less	113	40.0
2–4 times per month	72	25.5
2–3 times weekly	18	6.4
4 or more times per week	11	3.9

n = frequencies; % = percentage.

**Table 4:** Summary of the Alcohol Effects Questionnaire.

Measure	*n*	M	SD	α
Alcohol Effects	40	12.75	9.677	0.936
Questionnaire				
Alcohol Expectancy (Pos)	31	9.70	7.590	0.922
Alcohol Expectancy (Neg)	9	3.06	2.783	0.837

*n* = frequencies; M = mean; SD = Standard deviation; α = Cronbach’s alpha.

**Table 5:** Summary of the Spheres of Control Scale.

Measure	*n*	M	SD	*α*
Spheres of Control	30	131.66	20.890	0.825
Personal Efficacy Subscale	1–10	48.53	8.225	0.696
Interpersonal Control Subscale	11–20	20.59	7.022	0.707
Sociopolitical Control Subscale	21–30	32.65	8.127	0.697

*n* = frequencies; M = mean; SD = Standard deviation; α = Cronbach’s alpha.

**Table 6:** Alcohol consumption regressed on expectancies and self-efficacy.

Model		*r^2^*	B	*β*
Step 1				
	Positive Expectancy		0.041	0.33 **
	Negative Expectancy		0.022	0.07
Step 2				
	Interpersonal Control		0.020	0.16 *
	Sociopolitical Control		−0.003	−0.02
	Personal Efficacy		−0.002	−0.02
Total *r^2^*		0.187 (Adj. *r^2^* = 0.170)		

*r*^2^ = *r* squared; Adj. *r* ^2^ = adjusted r squared; B = coefficient; ***β*** = Beta. * *p* ≤ 0.05. ** *p* ≤ 0.01.
